# Genome-wide association analysis of time to heading and maturity in bread wheat using 55K microarrays

**DOI:** 10.3389/fpls.2023.1296197

**Published:** 2023-12-01

**Authors:** Yindeng Ding, Hui Fang, Yonghong Gao, Guiqiang Fan, Xiaolei Shi, Shan Yu, Sunlei Ding, Tianrong Huang, Wei Wang, Jikun Song

**Affiliations:** ^1^ Institute of Grain Crops, Xinjiang Academy of Agricultural Sciences, Urumqi, Xinjiang, China; ^2^ Institute of Crop Variety Resources, Xinjiang Academy of Agricultural Sciences, Urumqi, Xinjiang, China; ^3^ College of Agriculture, Xinjiang Agricultural University, Urumqi, Xinjiang, China; ^4^ Department of Computer Science and Information Engineering, Anyang Institute of Technology, Anyang, China; ^5^ Cotton Research Institute, Chinese Academy of Agricultural Sciences, Anyang, China

**Keywords:** wheat, heading, maturity, GWAS, candidate genes

## Abstract

To investigate the genetic mechanisms underlying the reproductive traits (time to flowering and maturity) in wheat and identify candidate genes associated, a phenotypic analysis was conducted on 239 wheat accessions (lines) from around the world. A genome-wide association study (GWAS) of wheat heading and maturity phases was performed using the MLM (Q+K) model in the TASSLE software, combined with the Wheat 55K SNP array. The results revealed significant phenotypic variation in heading and maturity among the wheat accessions across different years, with coefficients of variation ranging from 0.96% to 1.97%. The phenotypic data from different years exhibited excellent correlation, with a genome-wide linkage disequilibrium (LD) attenuation distance of 3 Mb. Population structure analysis, evolutionary tree analysis, and principal component analysis indicated that the 239 wheat accessions formed a relatively homogeneous natural population, which could be divided into three subgroups. The GWAS results identified a total of 293 SNP marker loci that were significantly associated with wheat heading and maturity stages (P ≤ 0.001) in different environments. Among them, nine stable SNP marker loci were consistently detected in multiple environments. These marker loci were distributed on wheat chromosomes 1A、1B、2D、3A、5B、6D and 7A. Each individual locus explained 4.03%-16.06% of the phenotypic variation. Furthermore, through careful analysis of the associated loci with large phenotypic effect values and stable inheritance, a total of nine candidate genes related to wheat heading and maturity stages were identified. These findings have implications for molecular marker-assisted selection breeding programs targeting specific wheat traits at the heading and maturity stages. In summary, this study conducted a comprehensive GWAS of wheat heading and maturity phases, revealing significant associations between genetic markers and key developmental stages in wheat. The identification of candidate genes and marker loci provides valuable information for further studies on wheat breeding and genetic improvement targeted at enhancing heading and maturity traits.

## Introduction

Wheat is one of the most important grain crops in the world and the third largest grain crop in China, and its yield plays a crucial role in China’s food security (Cheng et al., 2023). Different reproductive stages can reflect the growth and development rate and stability of wheat, and have a close relationship with maturity, yield and disease resistance of wheat, and have a great influence on the planting environment, regional planning, and the selection of varieties and cultivation and management measures of wheat, which has been a long-term concern of breeders (Hoogendoorn, 1985; Xu et al., 2022). Quality seedling emergence plays an important role in the yield of the subsequent crop, and the appropriate heading stage can ensure high and stable yield of wheat. The maturity stage of wheat controls the growth cycle of the crop’s reproductive period and provides a new direction for the selection of accessions. In addition, unfavorable climate change, shortage of natural resources and the threat of pathogenicity of pests and diseases in recent years have provided new challenges to wheat yield and quality as well as to the growth cycle of wheat (Morales et al., 2020).

A large number of studies have shown that reproduction period is controlled by multiple genes and a typical quantitative trait and that is susceptible to environmental influences. Mining gene loci associated with reproductive traits is an important basis for molecular marker-assisted breeding and interpretation of gene effects (Mackay and Powell, 2007). In recent years, with the emergence of new sequencing technologies, the reduction of sequencing costs and the release of wheat reference genome information, the development and application of wheat SNP microarrays have become more popular, and a large number of genetic loci controlling reproductive traits (time to flowering and maturity) have been excavated at home and abroad.

Nine QTLs located on 2D, 3B and 3D chromosomes were detected, with the highest contribution rate of 22.91% to heading stage (Song et al., 2006). A total of 9 heading stage related QTLs were detected on chromosomes 1B, 2B, 4B, 5A, 5B and 7B, which could explain 4. 55%-13. 40% of phenotypic variation (Wang et al., 2020). Wheat heading stage conformed to the genetic model of a pair of main genes + multiple genes through a multi-generation joint analysis (Wang et al., 2007). In recent years, there have been more reports on the studies of analyzing and locating QTL for wheat heading stage. A series of heading QTLs located on chromosomes 1A, 2B, 4D, 4BS, 5AL, 5DL, 7BS, 2D, 5A, 4A, 2A, and 2D were detected by DH populations (Sourdille et al., 2000). Flowering is a more water-sensitive period in wheat, which directly affects wheat yield. Two flowering QTLs located on chromosomes 1B and 1D under drought stress were detected through DH population, explaining 12.35% and 10.79% of the phenotypic variation, respectively, and two flowering QTLs located on chromosomes 1D and 5B under normal irrigation conditions, explaining 9.11% and 9.65% of the phenotypic variation, respectively (Yan et al., 2015). Five stable QTLs were detected on chromosomes 2A, 5B, 6B, 7A and 7D by genotyping RIL population with 90K chip, among which *QHd.cau-7D* can explain 29.35%-41.96% of phenotypic variation (Chen et al., 2020). Although a large number of wheat heading and anthesis loci have been reported, few of them have been used for breeding selection, and the loci controlling heading and anthesis differ from each other and the mechanism of inheritance is complex, with materials of different genetic backgrounds carrying different resistance factors. In this study, 239 wheat accessions (lines) were identified at heading and maturity stages, and genome-wide linkage analyses were carried out with SNP markers, in order to provide references for the study of genetic mechanisms of heading and maturity stages and molecular breeding of wheat.

## Materials and methods

### Materials

A total of 239 natural populations consisting of wheat breeding accessions and foreign introduced accessions promoted in winter wheat areas of China were used as test materials (**Table S1**). Among them, 213 materials were from China, including 6 from Anhui, 34 from Beijing, 19 from Hebei, 19 from Henan, 5 from Jiangsu, 11 from Shandong, 1 from Shanxi, 3 from Shaanxi, 1 from Tianjin and 115 from Xinjiang. There were 26 foreign materials, including 25 from the USA and 1 from Ukraine. All these materials could grow and develop normally in the test site.

### Experimental design

The experiment was conducted from September 2019 to July 2022 at Zepu Breeding Base (77°16’17.22 “N, 38°11’21.65 “E) of Xinjiang Academy of Agricultural Sciences (XAS) and in June 2021 at Anningqu Base (43°58’53.38 “N, 87°30’17.72 “E) of XAS. Where Xinjiang Zepu in 2020 was E1, Xinjiang Zepu in 2021 was E2, Xinjiang Anningqu in 2021 was E3, and Xinjiang Zepu in 2022 was E4. The three-year experiments at the four environments were conducted in a randomized block design with two rows of each material, row length of 1 m, row spacing of 0.2 m, and seeding of about 525 grains per square meter in a north-south row orientation. Light management, water and fertilizer management, water management and other field management were carried out according to normal management, each with three replications. The farming conditions and production conditions of each replication were the same.

### Determination of main reproductive traits (time to flowering and maturity)

Wheat plant samples were collected during several developmental stages, at seedling emergence, heading stage and maturity stage respectively. When more than half of the first true leaves of a variety were exposed to 2-3 cm above the ground surface and more than 50% of the wheat seedlings in the field reached the standard time, it was the seedling emergence of the variety, and the seedling emergence period was the number of days from sowing to seedling emergence; the middle part of the young spike of the plant was exposed to the scabbard leaf sheaths as the standard of heading, and the heading period was the number of days from sowing to heading; the maturity period of wheat was the milky ripening period, and the standard was that the stalks and leaves were yellowish-green, and the kernels were milky with milky contents. The maturity period of wheat was recorded at the stage of milky ripening when the stalks and leaves were yellow-green, the kernels had milky inclusions, the kernels turned yellowish at the end of milky ripening, the water content of the stalks was 65%-75% and more than half of the accessions fulfilled the criterion, and the maturity period was the number of days from sowing to maturity. From seedling to heading stage (S1) and from seedling to maturity stage (S2) were calculated from the recorded data in days.

### Methods of phenotypic analysis

The process of phenotyping was carried out by analysis of variance (ANOVA) as well as distributional evaluation of phenotypes, significance test of difference and correlation analysis, in addition all statistical analyses of data were implemented in IDE Spyder under Anacondas3 using Python 3.8.8 for data processing on a computer with an Intel i7-6800 K 3.40 GHz CPU, 16 GB of RAM, and an Nvidia GeForce GTX 2080Ti on a graphics workstation running the Win10 operating system. Statistical analyses, correlation analyses and tests of significance of differences were performed on wheat photosynthetic traits using the application software Pandas 1.3.2, Matplotlib 3.4.2, Scikit-Learn 0.24.2 and SPSS 21.0.

### Chip typing and population structure analysis

The kernel DNA was extracted using the SDS method (Wang et al., 2014). The DNA quality was assessed by 1.2% agarose gel electrophoresis, and the DNA concentration was measured with a NanoDrop™ND-2000 spectrophotometer (Wang et al., 2014), and 239 wheat materials were scanned using Affymetrix Axiom 55K array (Beijing Boao Jingdian Biotechnology Co., Beijing, China). Illumina’s Genome Studio Software was used for the original SNP typing of the samples. Markers with a filtration deletion rate of more than 20% and a minimum allele frequency (MAF) of less than 5%. High-quality SNP markers were retained for subsequent analysis (**Figure S1**).

The 2000 SNP markers after screening by random selection were screened for SNP markers that required a gene frequency greater than 10% and were evenly distributed on each different chromosome. Population structure analysis was performed by Structure v2.3.4 software (Zhu et al., 2008). The software was set up with reference to previous studies, and the results of the population structure were first presented visually as structure analysis plots through Tassel 5.0. Then the principal component analysis and Neighbor-jointing (NJ) evolutionary tree (Pritchard et al., 2000; Breseghello and Sorrells, 2006) were estimated for the population structure through Tassel 5.0, and finally plotted through the Matplotlib package for Python.

### Linkage disequilibrium calculation

The squared correlation coefficient between loci (r^2^) was used as a parameter to measure the linkage disequilibrium between two polymorphic loci between populations. r^2^ was mainly calculated using Tassel 5.0 software and the 95th percentile of the r^2^ value was used as a threshold to estimate the LD decay distance. The r^2^ values between chained clusters were considered for square root transformation to account for the effect of the background of chained imbalances between chained clusters. Parameter values greater than the 95% of this distribution were used as thresholds to intercept the LD decay distance within the same chained cluster. Therefore, during chain analysis, the physical distance between loci was compared and when the physical distance was less than that LD decay distance, the loci were considered to be the same locus (Yang et al., 2007).

### Association mapping

Association analysis, also known as linkage mapping or linkage disequilibrium mapping (LD mapping), is a quantitative genetic analysis technique that identifies loci or markers associated with a target trait based on the LD between alleles in different locis in a natural population by linking the diversity of the target trait to polymorphisms in the gene or marker.

In this study, we used Tassel v5.0 software combined with data for reproductive trait test and 55K SNP microarray data to carry out under different several reproductive stages and different water and drought treatments in wheat, and selected mixed linear model (MLM) for association analysis of the population. Through the analysis of the results calculated by the software, it is easy to see that the SNP marker can be identified as significantly associated with the trait when P ≤ 0.001. Manhattan and QQ plots can be drawn from the results. The QQ plot can be used to further judge the correctness of the results of the association analysis and to exclude the appearance of some false positives. The horizontal coordinate of the Manhattan plot is the 21 chromosomes of wheat, and the vertical coordinate is the negative logarithm of the P-value of SNP markers. The distribution of SNP markers in all chromosomes and the loci of significant association can be seen through the Manhattan plot (Maccaferri et al., 2016).

### Candidate gene prediction

The extended sequences of the stable SNP markers were subjected to BLAST comparison in the common wheat Chinese Spring genome database (https://urgi.versailles.inrae.fr/blast_iwgsc/) and gene function annotation in the Wheat Omics 1.0 database (http://wheatomics.sdau.edu.cn/) for gene function annotation.

## Results

### Analyses of phenotypic variation during the reproductive period

**Figure 1:**
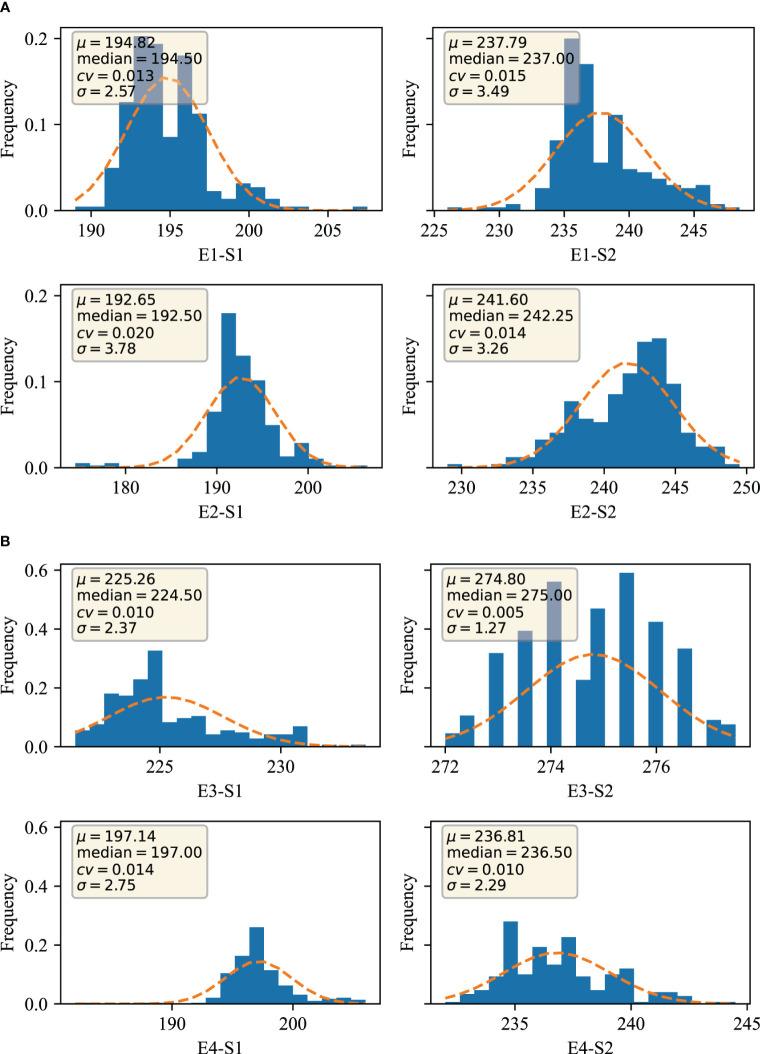
Distribution of traits in different environments **(A)** Germination-spike and germination-maturity in E1 and E2 environments; **(B)** Germination-spike and germination-maturity in E3 and E4 environments. E1: 2020 Zepu, Xinjiang; E2: 2021 Zepu, Xinjiang E3: 2021 Anning Drain, Xinjiang E4: 2022 Zepu, Xinjiang; S1: heading stage; S2: maturity stage.

Two traits at S1 and S2 were obtained for phenotyping at four environments, E1, E2, E3 and E4, respectively. The data were evaluated through four dimensions, which are mean expressed as μ, median expressed as median, coefficient of variation expressed as cv (coefficient of variation), and standard deviation expressed as σ. From **Figure 1**, it can be seen that the trait µ of S1 stage in E1 environment was 194.82, median was 194.50, cv was 1.30% and σ was 2.57; the trait µ of S2 stage in E1 environment was 237.79, median was 237.00, cv was 1.50% and σ was 3.49; the trait of S1 stage in E2 environment µ was 192.65, median was 192.50, cv was 2.00%, and σ was 3.78; S2 trait µ was 241.60, median was 242.25, cv was 1.50%, and σ was 3.49 in E2 environment; and S1 trait µ was 225.26 in E3 environment, median was 224.50 with a cv of 1.00% and a σ of 2.37; the S2 trait µ in the E3 environment was 274.80 with a median of 275.00, a cv of 0.50%, and a σ of 1.27; the S1 trait µ in the E4 environment was 197.14 with a median of 197.00, a cv of 1.40%, and a σ of 2.75; the E4 environment the S2 trait µ was 236.81, median was 236.50, cv was 1.00%, and σ was 2.29. Overall the S1 trait µ was 192.65-225.26, median was 192.50-224.50, cv was 1.00%-2.00%, and σ was 2.37-3.78; the S2 trait µ was 236.81-274.80, median was 236.50-275.00, cv was 0.50%-1.50%, and σ was 1.27-3.49. The data in different environments showed a continuous and normal distribution, which is in line with typical quantitative trait characteristics.

By further analyzing the variance of the S1 and S2 traits of winter wheat obtained from different environments, **Table 1** shows that the standard deviation ranges from 1.27-3.78, and analyses from the point of view of the analysis of variance indicate that the S1 and S2 traits of wheat are mainly genotypically determined, and also affected by the environment, and that the genetic factor is the main reason for its phenotypic variability.

**Table 1:** Descriptive statistics of wheat reproductive traits (time to flowering and maturity).

Environments	Traits	Max	Min	Mean	Standard Deviation	Cv (%)
E1	S1	207.5	189	194.83	2.57	1.32
	S2	248.5	226	237.79	3.5	1.47
E2	S1	206.5	174.5	192.65	3.78	1.96
	S2	249.5	229	241.6	3.27	1.35
E3	S1	233.5	221.5	225.26	2.37	1.05
	S2	277.5	272	274.8	1.27	0.46
E4	S1	206	182	197.14	2.75	1.4
	S2	244.5	232	236.81	2.3	0.97

### Correlation analyses of reproductive traits (time to flowering and maturity)

**Figure 2:**
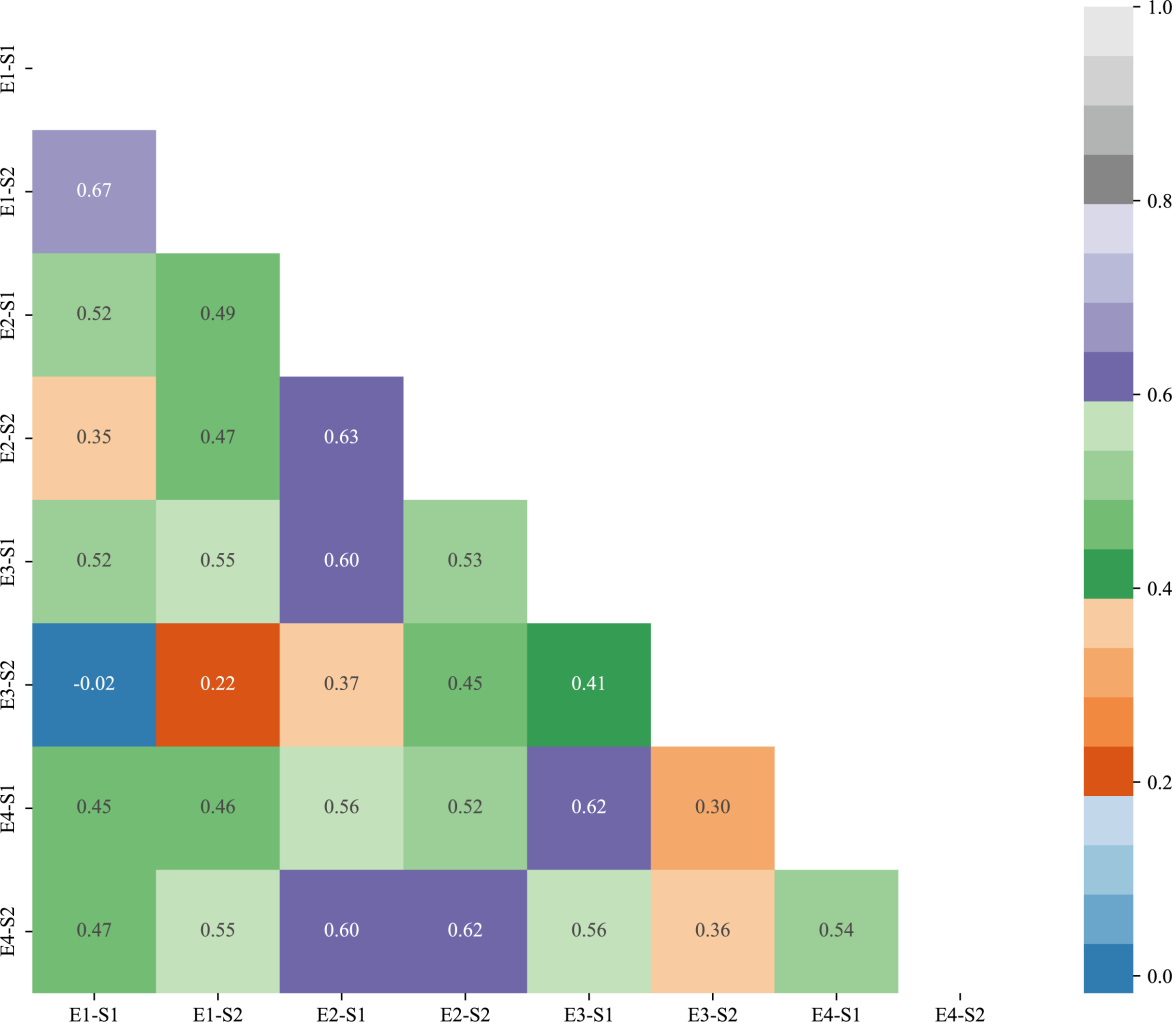
Correlation plots of germination-sprouting and germination-maturity traits in different environments.

The results of correlation analysis of two traits at S1 and S2 stage at four environmental points, E1, E2, E3 and E4, are shown in **Figure 2**. The correlations of reproductive traits of winter wheat in different environments were compared in the figure, from which it can be seen that most of the winter wheat reproductive traits reached the highly significant level (p<0.001).The correlations of S1 stage traits in E1 environment were -0.02-0.67, and the correlations of S2 stage traits in E1 environment were 0.22-0.55; and the correlations of S1 stage traits in E2 environment were 0.37-0.55; and the correlations of S2 stage traits in E3 and E4 environments were 0.37-0.55. correlation was 0.37-0.63 for S1 stage trait in E2 environment and 0.45-0.62 for S2 stage trait in E2 environment; correlation of S1 stage trait in E3 environment was 0.41-0.62 and correlation of S2 stage trait in E4 environment was 0.36-0.62. Correlation of S1 stage trait in different environments was 0.45-0.63 and correlation of S2 stage trait in different environments was 0.45-0.63. Overall the correlation of S1 and S2 of bread wheat in different environments from 0.13-0.81, reaching highly significant levels (p<0.001).

### Population structure and evolutionary tree analysis

**Figure 3:**
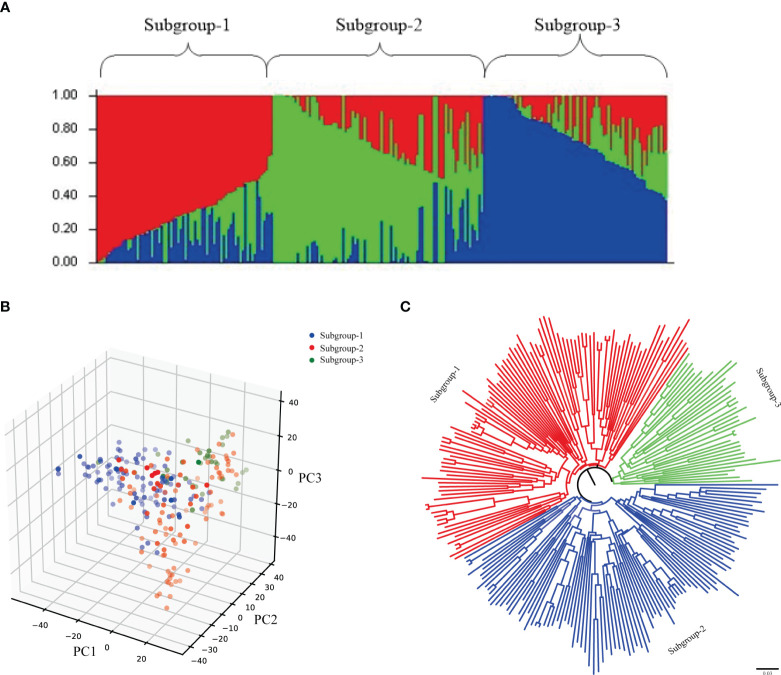
Population structure analysis of 239 wheat accessions. **(A)** Population structure analysis; **(B)** Neighbor-joining method evolutionary tree; **(C)** Principal component analysis.

Using Structure software to analyze the population structure of 239 test materials (**Figure 3**), the group structure was divided by group structure, evolutionary tree and principal component analysis. The results showed that the results of the three analysis methods were consistent, and it was reasonable to divide the whole population into three subgroups, of which subgroup 1 had 95 accessions (lines), subgroup 2 had 89 accessions (lines), and subgroup 3 had 55 accessions (lines). The distribution frequencies of the materials contained in the three subgroups were 39.75%, 37.24% and 23.01% in the following order. The LD decay distances of 239 wheat accessions (lines) in genomes A, B, D and the whole genome were calculated to be 3, 3, 2 and 3 Mb, respectively. Based on the LD decay distances of the whole genome, the loci within the interval of 3 Mb before and after the physical map were identified as a candidate locus.

### GWAS analysis of reproductive traits (time to flowering and maturity)

**Figure 4:**
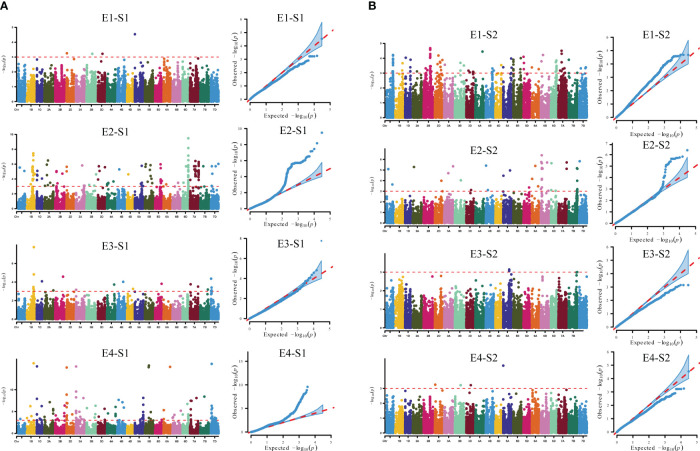
GWAS of reproductive traits in different environments. **(A)** Manhattan and quantile-quantile plots for S1 in different environments. **(B)** Manhattan and quantile-quantile plots for S2 in different environments. E1: 2020 Zepu, Xinjiang; E2: 2021 Zepu, Xinjiang E3: 2021 Anning Drain, Xinjiang E4: 2022 Zepu, Xinjiang; S1: heading stage; S2: maturity stage.

The S1 and S2 of 239 wheat accessions (lines) were combined with 16,649 high-quality SNP markers typed by 55K SNP chip screening for genome-wide association analysis using TASSEL 5.0 software. Based on the MLM (Q+K) model, the markers were considered to be significantly associated with the trait when P ≤ 0.001, and loci detected in multiple environments were considered to be stably heritable (**Figure 4**, **Table 2**, **Table S2**). Analysis of the GWAS results showed that a total of 238 SNP markers were detected for the S1 trait, of which a total of eight markers were detected in multiple environments, distributed on chromosomes 1B、2D、3A、5B、6D、7A、7D with individual interpretable phenotypic variation rates of 4.03%-16.06%. *AX-109375483* and *AX-110425403* located on chromosome 1B, were both detected simultaneously in both environments, with phenotypic variation rates of 4.49%-4.96% and 4.82%-16.06%, while *AX-108940388* located on chromosome 2D, was detected simultaneously in both environments E2 and E4, with phenotypic variation rates of 5.03%-15.08%; *AX-110591324* located on chromosome 3A was detected in both E2 and E4 environments at the same time with a phenotypic variation rate of 5.16%-15.31%; *AX-109429484* located on chromosome 5B was detected in both E2 and E4 environments at the same time with a phenotypic variation rate of 4.03%-15.53%; *AX-111919223* located on chromosome 6D, was detected in both E2 and E4, with a phenotypic variation rate of 5.20%-6.73%; *AX-110961085* located on chromosome 7A, was detected in both E2 and E4, with a phenotypic variation rate of 4.14%-8.09%; and *AX-108866484* located on chromosome 7D, was detected in both E2 and E4, with a phenotypic variation rate of 4.14%-8.09%. *AX-108866484* located on chromosome 7D was detected in both E3 and E4 environments, with a phenotypic variation rate of 4.36%-15.91%, respectively. A total of 55 SNP markers were detected for the S2 trait, of which *AX-110986688* located on chromosome 1A was detected in both E1 and E2 environments, with a single locus explaining the phenotypic variation rate of 4.18%-5.08%.

**Table 2:** Information of significantly associated loci of wheat reproductive traits (time to flowering and maturity).

Traits	Marker	Chr.	Position (Mb)	P-value	*R^2^* (%)	Environment
S1	*AX-109375483*	1B	535.23-536.57	1.10E-05-3.21E-05	4.49-4.96	E2、E3
	*AX-110425403*	1B	581.10-587.17	8.792E-17-1.50E-05	4.82-16.06	E3、E4
	*AX-108940388*	2D	12.92-17.45	8.38E-16-9.26E-06	5.03-15.08	E2、E4
	*AX-110591324*	3A	16.63-20.15	4.86E-16-6.82E-06	5.16-15.31	E2、E4
	*AX-109429484*	5B	466.61-474.97	2.97E-16-9.37E-05	4.03-15.53	E2、E4
	*AX-111919223*	6D	466.89-472.91	1.87E-07-9.68E-06	5.20-6.73	E2、E4
	*AX-110961085*	7A	730.53-735.46	8.14E-09-3.12E-06	4.14-8.09	E2、E4
	*AX-108866484*	7D	57.69-59.71	1.24E-16-4.36E-05	4.36-15.91	E3、E4
S2	*AX-110986688*	1A	542.72-543.95	8.24E-06-6.61E-05	4.18-5.08	E1、E2

### Functional prediction of candidate genes for reproductive traits (time to flowering and maturity)

SNP markers with large phenotypic effect values that could be stably inherited were searched in the Chinese Spring Genome Database of common wheat and BLASTx sequence comparison was performed in the NCBI database, and a total of nine candidate genes most likely to be associated with reproductive traits were mined (**Table 3**).

**Table 3:** Information of candidate genes for wheat reproductive traits (time to flowering and maturity).

Maker	Chr	Physical location (Mb)	Genes	Gene annotation
*AX-109375483*	1B	535.23-536.57	*TraesCS1B01G312100*	Glycosyltransferase
*AX-110425403*	1B	581.10-587.17	*TraesCS1B01G356000*	F-box protein
*AX-108940388*	2D	12.92-17.45	*TraesCS2D01G044700*	Cytochrome P450
*AX-110591324*	3A	16.63-20.15	*TraesCS3A01G036000*	Zinc finger family protein
*AX-109429484*	5B	466.61-474.97	*TraesCS5B01G288700*	S-acyltransferase
*AX-111919223*	6D	466.89-472.91	*TraesCS6D01G404800*	Calcium-dependent protein kinase
*AX-110961085*	7A	730.53-735.46	*TraesCS7A01G560200*	Photosystem II stability/assembly factor HCF136
*AX-108866484*	7D	57.69-59.71	*TraesCS7D01G098100*	Zinc transporter
*AX-110986688*	1A	542.72-543.95	*TraesCS1A01G362500*	Cytokinin riboside 5’-monophosphate phosphoribohydrolase

Candidate genes for reproductive traits (time to flowering and maturity) are mainly associated with photosynthesis, Ca^2+^ transport, phytohormone biosynthesis and signal transduction in crops. The genes *TraesCS1B01G312100* and *TraesCS1B01G356000* located on chromosome 1B encode glycosyltransferases and F-box family proteins, respectively; *TraesCS2D01G044700* located on chromosome 2D is associated with cytochrome proteins; *TraesCS3A01G036000* on chromosome 3A encodes a zinc finger family protein; *TraesCS3A01G03600* on chromosome 3A encodes a zinc finger family protein; and *TraesCS3A01G036000* encodes a zinc finger family protein; *TraesCS5B01G288700*, located on chromosome 5B, encodes an S-acyltransferase; *TraesCS6D01G404800*, located on chromosome 6D, encodes a calcium-dependent protein kinase; *TraesCS7A01G404800* located on chromosome 7A, encodes a calcium-dependent protein kinase; and *TraesCS7A01G560200* encodes Photosystem II stability/assembly factor HCF136; *TraesCS7D01G098100* on chromosome 7D encodes zinc finger protein; *TraesCS1A01G362500* on chromosome 1A encodes Cytokinin riboside 5’-monophosphate phosphoribohydrolase.

## Discussion

### Time to heading and maturity trait association analyses

With the rapid development of biology and bioinformatics, GWAS analysis has become an important way to study quantitative traits in plants, and the mining of genes related to reproductive traits (time to flowering and maturity) in wheat has been promoted to a greater extent. Meanwhile, reproductive traits are typical quantitatively inherited traits, which are subject to the joint action of genotype and environment. In this study, through four environmental sites and three years of data accumulation, a total of nine stable genetic loci in multiple environments were excavated to be significantly or very significantly associated with wheat heading and maturity stages, which affect wheat adaptation and yield and are complex quantitative traits regulated by multiple genes. It has been shown that the genes associated with heading and maturity stages are mainly located on chromosomes 1A、2A、 2B、2D、3A、5A、5B、5D and 7B (Pritchard et al., 2000; Somers et al., 2004; Evanno et al., 2005; Liu and Muse, 2005; Jingna et al., 2014; Shi et al., 2019). Some SNP loci on chromosomes 1B、3D and 7D were identified by GWAS analysis as significantly associated with heading (Hardy and Vekemans, 2002; Zou et al., 2017; Ma et al., 2021; Zhang et al., 2021). In this study, we identified that the SNPs significantly associated with heading were mainly located on chromosomes 1A、1B、2D、3A、5B、6D and7A, and the results were more consistent with the previous localization results.

### Functional analysis of candidate genes

The GWAS was used to detect 9 SNP marker locis that were significantly associated with reproductive traits (time to flowering and maturity) in wheat, and nine candidate genes that might be related to reproductive traits were screened in the Chinese Spring Genome Database of common wheat. The genes *TraesCS1B01G312100* and *TraesCS1B01G356000* located on 1B encode glycosyltransferases and F-box family proteins, respectively; glycosylation is an important post-translational modification of proteins in plants, which is involved in the regulation of various biological functions. Glycosyl transferase (GT) is one of the most important enzymes in the class of glycosylation enzymes. F-box family proteins have important roles in physiological processes such as phytohormone signaling, light signaling and floral organ development (Bi et al., 2006). The gene *TraesCS2D01G044700*, located on 2D, is related to cytochrome proteins; cytochrome P450 is an important oxidase in the microsomal mixed-function oxidase family, which is widely distributed in living organisms, and is involved in the synthesis and metabolism of a wide range of endogenous and exogenous compounds, which have important functions in biological oxidation, nitrogen fixation, photosynthesis, energy conversion, and storage (Himi et al., 2011). *TraesCS3A01G036000* and *TraesCS7D01G098100* located on chromosomes 3A and 7D encode zinc finger family proteins; which can be involved in physiological and biochemical regulatory mechanisms in plants during growth and development. *TraesCS5B01G288700* located on chromosome 5B encodes S-acyltransferase, which is a metabolite in plants that has an important role in growth and development and under drought stress. The gene *TraesCS6D01G404800* located on 6D encodes calcium-dependent protein kinase; this enzyme acts as a cellular second messenger, Ca^2+^ coordinates the perception of various physiological responses in plants, and the Ca^2+^ sensor transmits calcium signals downstream and triggers a cascade of reactions, regulating the processes of plant growth, development and response to the environment. The gene *TraesCS7A01G560200* on chromosome 7A encodes Photosystem II stability/assembly factor HCF136; PS II is a pigmented protein complex present in the membranes of cysts in plants that drives the light-activated transfer of electrons from water to plastocysts, accompanied by the production of molecular oxygen. The gene *TraesCS1A01G362500* located on chromosome 1A encodes Cytokinin riboside 5’-monophosphate phosphoribohydrolase. Cytokinins are a class of N6-adenine analogues, which are closely related to crop yield and plays a key role in the regulation of plant growth and development, including the promotion of fruiting, the release of apical dominance, the promotion of cell division, and the shortening of the transition from nutrient growth to reproductive growth. The analysis of gene function lays the foundation for our next step of functional marker development.

## Conclusion

In this study, a comprehensive genome-wide association analysis was conducted on 239 wheat accessions (lines) from both domestic and international sources. The analysis focused on the heading and maturity stages of wheat using a 55K SNP microarray and a Q+K mixed linear model. The aim was to identify SNP markers associated with these important developmental phases.

The analysis identified a total of 293 SNP marker loci that showed significant associations (*P ≤ 0.001*) with heading and maturity stages in wheat. Among these markers, nine were found to be consistently associated with these traits in multiple environments, indicating their stability and reliability. These stable SNP marker loci were located on different chromosomes of wheat, including 1A、1B、2D、3A、5B、6D and 7A. Furthermore, the researchers investigated the phenotypic effect values and inheritance patterns associated with these SNP markers. By searching the Chinese Spring Genome Database of common wheat, we identified nine candidate genes that were most likely to be associated with the heading and maturity stages of wheat. These candidate genes were selected based on their significant phenotypic effect values and stable inheritance patterns. It is important to note that the specific details of the candidate genes were not provided in the information provided. However, the identification of these candidate genes suggests their potential involvement in regulating the heading and maturity phases of wheat development.

## Data availability statement

The datasets generated during and/or analyzed during the current study are available from the corresponding author upon reasonable request.

## Author contributions

YD: Conceptualization, Funding acquisition, Resources, Supervision, Writing – original draft, Writing – review & editing. HF: Conceptualization, Funding acquisition, Resources, Supervision, Writing – original draft, Writing – review & editing. YG: Conceptualization, Funding acquisition, Resources, Supervision, Writing – original draft, Writing – review & editing. GF: Formal Analysis, Writing – original draft, Writing – review & editing. XS: Formal Analysis, Writing – original draft, Writing – review & editing. SY: Data curation, Investigation, Methodology, Writing – review & editing. SD: Data curation, Investigation, Methodology, Writing – review & editing. TH: Data curation, Investigation, Methodology, Writing – review & editing. WW: Data curation, Investigation, Methodology, Writing – review & editing. JS: Data curation, Investigation, Methodology, Writing – review & editing.

## Supplementary material

Supplementary Figure 1 Density distribution of SNPs on chromosomes.
