# Supplementary figures and images for: Genome-wide association analysis of time to heading and maturity in bread wheat using 55K microarrays
Source: Front Plant Sci. 2023 Dec 1;14:1296197. doi: 10.3389/fpls.2023.1296197 (PMC10722194; doi:10.3389/fpls.2023.1296197)

# The number of SNPs within 1Mb window size

0Mb      92Mb      184Mb      276Mb      368Mb      460Mb      552Mb      644Mb      736Mb      828Mb

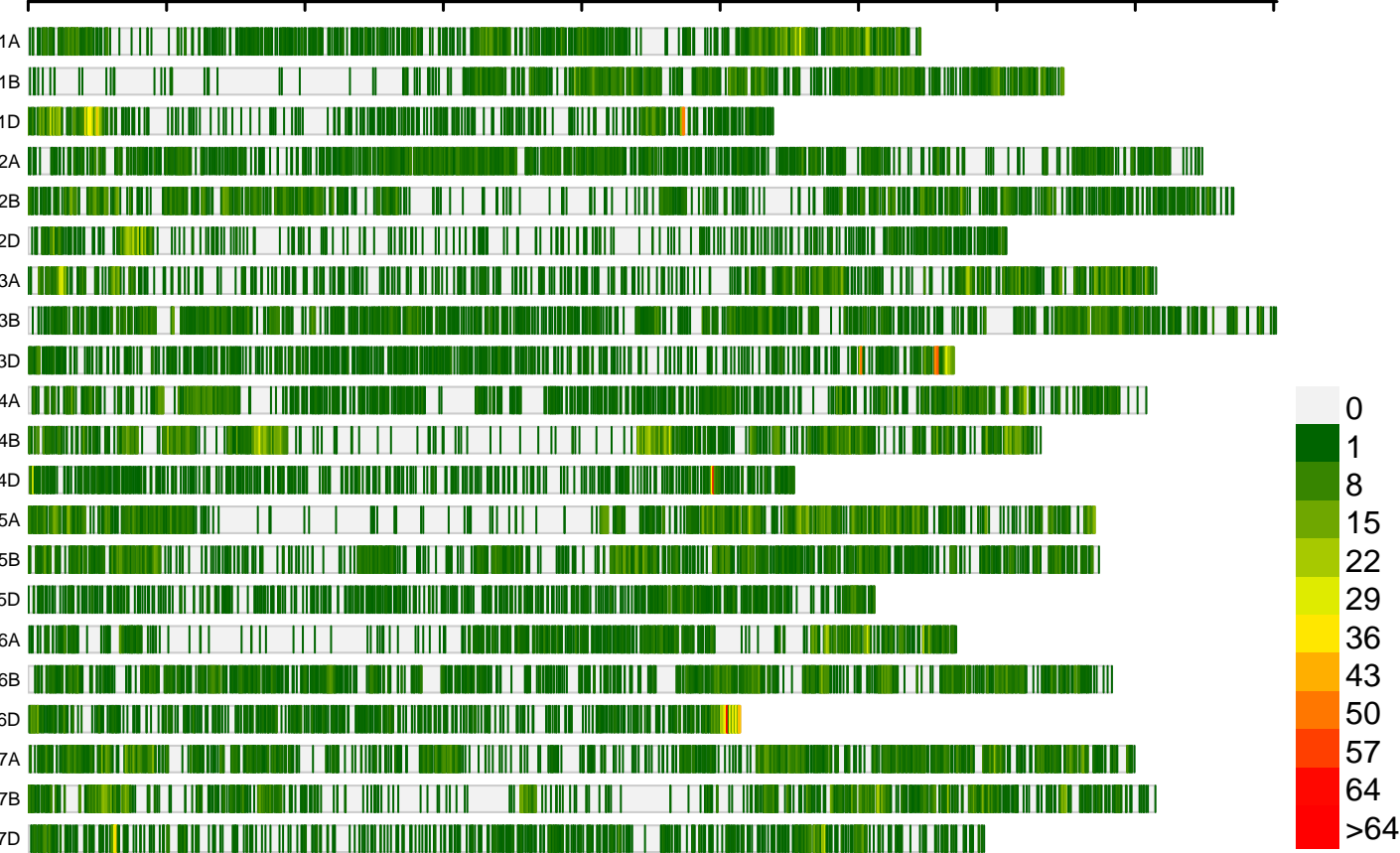

Supplement: Supplementary Figure 1 — Density distribution of SNPs on chromosomes. [file DataSheet_1.pdf]
